# Incidental Thoracic and Abdominal Findings in Diagnostic Imaging

**DOI:** 10.1155/2018/4051606

**Published:** 2018-02-12

**Authors:** Arnaldo Scardapane, Giuseppe Angelelli, Luca Macarini

**Affiliations:** ^1^Interdisciplinary Department of Medicine, Section of Diagnostic Imaging, Bari Medical School, Bari, Italy; ^2^Radiology Department, University of Foggia, Foggia, Italy

In recent years the rapid diffusion of advanced imaging studies such as magnetic resonance and multidetector computed tomography has resulted in a considerable increase of asymptomatic and unexpected findings. A recent meta-analysis by Lumbreras et al. showed that incidental findings are commonly encountered in diagnostic imaging with a mean frequency of 23.6% across all imaging modalities [1]. Therefore, the radiologist has more and more frequently the task of correctly interpreting these lesions and giving comprehensive information to the patients about their clinical relevance. The ability to correctly detect likely benign findings may help reduce unnecessary imaging studies, although the lack of established follow-up guidelines for many nonunivocal interpretation results suggests that further studies are needed.

This special issue of BioMed Research International reviews the most common incidental thoracic and abdominal findings recognized by any imaging technique (X-ray, ultrasound, MDCT, MRI, and interventional radiology procedures).

The use of cross-sectional cardiac imaging for the diagnosis of cardiovascular disease is continuing to increase [2–4]. Cardiac magnetic resonance imaging (cMRI) was recently proposed as a new noninvasive imaging modality that allows higher structural and functional assessment of the heart in any desired plane without radiation. A typical cMRI exam includes several structures besides the cardiovascular system, such as parts of lungs, thorax, and upper abdomen. In this special issue, M. Gravina et al. analyse retrospectively the prevalence and the nature of incidental extracardiac findings (IEFs) in a large series of patients referred for cMRI. The incidences of IEFs as well as their clinical management are discussed in detail.

In this issue, M. A. Mazzei et al. describe the prevalence, as incidental findings, and the underreporting rate of pleural plaques (PPs) in chest CT scans. As we know, PPs represent a risk factor for mortality from lung cancer in asbestos-exposed workers and they are often underreported in absence of clinical suspicion. This study shows that knowledge of the typical appearance and location of PPs is crucial for their correct recognition and their differential diagnosis.

Incidental renal masses are frequently encountered. In fact, it has been estimated that over half of patients over the age of 50 years harbour at least one renal mass, and often several are found during one radiologic examination [5, 6]. Most of these are benign simple cysts that can be definitely diagnosed as benign on the basis of cross-sectional imaging and do not require treatment. However, complex cystic and solid renal masses are also discovered, many of which are clearly malignant and need to be surgically removed, while others may not require surgical intervention. The original research report authored by S. Mazziotti et al. provides a practical guide to identify and classify the main incidental renal findings and their correct management is well detailed.

Incidental gastrointestinal findings are commonly detected on MDCT exams performed for various medical indications. As pointed out by the comprehensive review by G. Di Grezia et al. on the radiological appearances' spectrum of several gastrointestinal acute conditions in this issue, MDCT exam plays a crucial rule since an appropriate differential diagnosis is needed. Lastly the prevalence of incidental peritracheal cysts in association with lung fibrosis is discussed in a paper by H. Y. Kim et al.

In conclusion, the present special issue offers useful guides for the correct interpretation and management of the main incidental thoracic and abdominal findings encountered using cross-sectional imaging. Furthermore, on the basis of these considerations, these articles also emphasize the role of the radiologist as the only figure with the appropriate professional background for the interpretation of all the findings that can be unexpectedly encountered in complex and organ-tailored examinations and to provide the clinicians and patients with the right recommendations.

*Arnaldo Scardapane* *Giuseppe Angelelli* *Luca Macarini*
